# Histopathological changes in the head kidney induced by cadmium in a neotropical fish *Colossoma macropomum*

**Published:** 2013-12-25

**Authors:** R. Salazar-Lugo, A. Vargas, L. Rojas, M. Lemus

**Affiliations:** 1 *Departamento de Bioanálisis, Laboratorio de Proteínas e Inmunotoxicidad, Postgrado de Biología Aplicada, Escuela de Ciencias, Núcleo de Sucre, Universidad de Oriente, Cumaná, Venezuela*; 2 *Laboratorio de Histología, Instituto de Investigaciones en Biomedicina y Ciencias Aplicadas (IIBCA-UDO), Núcleo de Sucre, Universidad de Oriente, Cumaná, Venezuela*; 3 *Departamento de Biología, Laboratorio de Ecofisiología y Contaminación Ambiental, Escuela de Ciencias, Núcleo de Sucre, Universidad de Oriente, Cumaná, Venezuela*

**Keywords:** Cadmium, *Colossoma macropomum*, Granulocytes, Head kidney, Histology

## Abstract

We evaluated the effect of cadmium (Cd) on the structure and function of the head kidney in the freshwater fish *Colossoma macropomum* (*C. macropomum*). Juveniles were exposed to 0.1 mg/L CdCl_2_ for 31 days. Blood samples were examined using hematological tests and head kidney histology was determined by light microscopy. The concentration of Cd in the head and trunk kidneys was measured using an atomic absorption spectrophotometer. Cd produced histopathological changes in the head kidney, the most evident of these being: the thickening of the vein wall, an increase in the number of basophils/mast cells close to blood vessels and a severe depletion of hematopoietic precursors especially the granulopoietic series. In the blood, a decrease in the total leucocytes and hemoglobin concentration was observed. Cd-exposed fish showed higher Cd concentrations in the trunk kidney than the head kidney. In conclusion, exposure to Cd affected precursor hematopoietic cells in *C. macropomum*.

## Introduction

Cadmium (Cd) is a toxic heavy metal that has become increasingly important as an environmental hazard to both humans and wildlife. Cd can act as a mitogen, stimulates cell proliferation, inhibits apoptosis, inhibits DNA repair, and promotes cancer in a number of tissues (Satarug and Moore, 2012). In addition, it causes tissue damage, notably in the kidney, by inducing cell death. There is also evidence that environmental exposure to Cd affects renal function (Nordberg *et al.*, 2012). In the fish, *Dicentrarchus labrax* and *Centropomus undecimales*, Cd has been reported to induce histological and cytological changes to the kidneys (Giari *et al.*, 2006).

In Venezuela, moderate Cd concentrations in rivers and catchment basins have been reported (Vaquero *et al.*, 2004), with mining activity and the petroleum industry being the primary source. Moderate Cd concentrations in fish, including *Colossoma macropomum* (*C. macropomum*) have also been registered (Salazar-Lugo, 2009). This species is widely distributed in South America and is abundant in the catchment areas of the Amazon and Orinoco rivers where it constitutes an important component of the local riverside economy. Moreover, the cachama presents a number of characteristics that may make it an appropriate model that can be used as indicator species in biomonitoring programs.

Chronic Cd exposure in *C. macropomum* produces a decrease in blood reticulocytes and total leukocytes (Salazar-Lugo *et al.*, 2011) affecting its innate immune response. These observations suggest that Cd could be producing damage in leucopoietic organs such as the head kidney. This organ plays an important roles as main haematopoiectic, immune and endocrine organ in many fish; because of this, Cd accumulation in head kidney can produce toxic effects on many important physiological processes such as changing hormonal level, immunological mechanism or hematological parameters (Lafuente *et al.*, 2004; Dangre *et al.*, 2010; Salazar-Lugo *et al.*, 2011; Vargas *et al.*, 2012; Kondera and Witeska, 2013) affecting the fish survival.

In this study we examined histologically the effect of chronic Cd exposure on the head kidney of *C. macropomum* as well as evaluating some blood parameters used as indicators of fish health.

## Materials and Methods

### Fish maintenance

*C. macropomum* juveniles (87.69±34.23 g and 17.87±7.88 cm) were supplied by ALMACA Aquaculture (Punta de Mata, Venezuela) and maintained for 3 weeks in holding tanks with continuously aerated and flowing de-chlorinated tap water (pH 7.2 - 7.8) at a constant temperature of 28±1 ºC. Fish were fed *ad libitum* with food specific for *C. macropomum* (PURINA, Venezuela). Artificial illumination was reduced by surrounding the aquariums with transparent black bags so as not to disturb the natural condition of these fish because these fishes live in dark freshwater. Water, pH (7.3±0.05), temperatures (28±1 ºC), oxygen level (3.8 mg/L, Total Hardness (106 mg/L) were controlled during experiments.

### LC_50_ 96 h determination

For the experimental LC_50_ 96h protocol, ten (10) juvenile cachama were transferred to individual 40 L glass aquariums 48 h prior to the experiments. Five groups of fishes (with replicates) were exposed to 128, 64, 32, 16, 8, and 2 mg/L Cd for 96 h. Another group was kept in water free of Cd and was considered to be control fish. Mortality was determined at 12, 24, 36, and 96 h after exposure to Cd. The recorded data were analyzed using probit method (Stephan, 1977) with the help of EPA Probit Software 1.5 Version.

### Cd Bioassays

Fish (n = 48) were divided in two experimental groups: Control and Cd-exposed groups (12 fish per group with replicates) and placed in 16 L glass aquariums (six fish /aquarium). A sub lethal concentration of 0.1 mg/L Cd from CdCl_2_^.^H_2_O (Aldrich) was selected according to the LC_50_ at 96 h (<0.1% LC_50_ 96h). Fish were exposed for 31 days, using a semi-static test system.

Each aquarium was continuously aerated and the same physical and chemical characteristics of the water as those for the laboratory acclimation were maintained. The initial Cd concentration in the water was 0.85-0.95 mg/LCd; eighty percent (80%) of Cd aquarium water was renewed every day. Before water renewal, an aliquot was taken to measure the residual Cd concentration by spectrophotometer analysis (0.25 mg/LCd).

The fish were then fed and the remaining food was removed. Control fish were maintained under the same conditions in water devoid of detectable Cd. Fish were fed before adding Cd to the water (every 24 h). No fish mortality was observed during the experiment. Fish were sacrificed after 31 days of exposition and the head and trunk kidneys were dissected and weighed.

### Histological analyses

The histological analyses of head kidney were done according to Rojas *et al*. (1997). Fish (n = 6, 3 fish per group) were sacrificed and the kidney samples dissected at 21ºC. Each sample was cut into 2 mm^2^ sections and placed in a fixative (3% glutaraldehyde pH 8.0) for 2 h. They were then washed in a 0.1 M phosphate buffer (15 min), post-fixed in 1% OsO_4_ in a 0.1M phosphate buffer (1 h), rinsed in phosphate buffer followed by two baths in distilled water, dehydrated in a graded ethanol series and bathed in propylene oxide (10 min). The tissues were then successively infiltrated with a 2:1 mixture of Epon and propylene oxide for 6 h, and pure Epon-812 medium for another 2 h.

Finally, they were embedded in silicone rubber molds filled with Epon-812 and polymerized at 60°C for 48 h. Semi-thin (0.7 µm) sections were cut, mounted on glass slides and stained with toluidine blue. Observations and micrographics were made using a Zeiss photomicroscope, Axioscop FL40.

### Head kidney Imprints and Hematological analysis

Head kidney imprints were obtained by pressing tissue on the slides. Blood smears were prepared from peripheral blood obtained by puncture of the caudal vein. Imprints were air-dried, fixed in absolute methanol for three min at room temperature and stained with 10% McGruwall-Wrigh-Giemsa. The slides were examined under a Zeiss photomicroscope, Axioscop FL40.

Approximately, 1.5 mL of venous blood was drawn from each subject. Hemoglobin was determined with Drabkin’s reagent and absorbance was measured at 540 nm. Hematocrit was determined by the microhematocrit technique (Blaxhall and Daisley, 1973). Blood smears were fixed with methanol and stained with 10% McGruwall-Wrigh-Giemsa. The slides were examined under a Zeiss photomicroscope, Axioscop FL40.

### Cd Determination

The concentration of Cd in tissues and water was analyzed using a Flame Atomic Absorption Spectrophotometric (Perkin Elmer 3110). The quality of the heavy metal measurements was assured by the use of DORM-2 dogfish muscle; Certified Reference Material for Trace Metals (National Research Council of Canada).

The average values of the heavy metals obtained by the analytical process agreed with the certified values 90-97% (Sardiñas and García, 1996).

### Statistics

Results are expressed as mean ± standard error (SE) and the medians of the two groups (Cd-exposed and controls) were compared using the non-parametric Mann-Whitney (Wilcoxon) W test. Differences were considered significant at *P*<0.05. All statistical analysis was made with the program Stat graphics Plus Version 5.1 (Addlink Software Scientific, S.L.).

## Results

Analysis of data collected on percent mortality at 96 h exposure to different concentrations of Cd revealed the LC_50_ value to be at 38.47 mg/L. Fish showed dark skin and reduced movement. Nevertheless, the fish always stayed grouped.

**Fig. 1:**
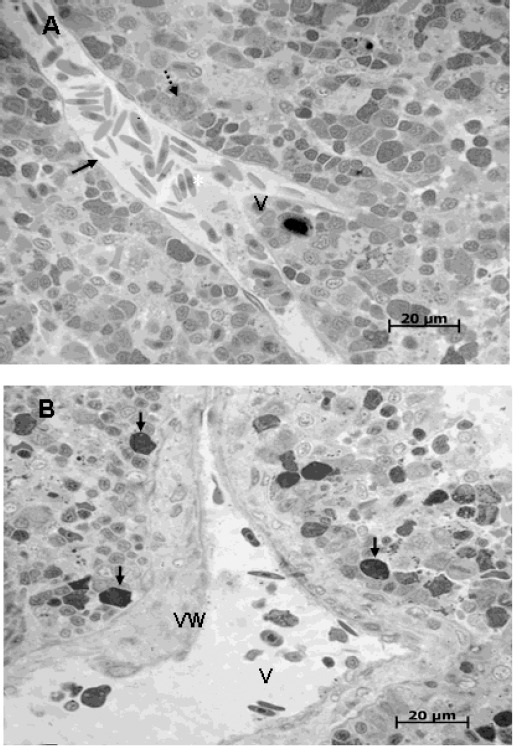
Cross-section of the head kidney of *C. macropomum*. **A:** head kidney from a control fish showing a characteristic parenchyma; vein wall (black arrow), vessel (V), erythrocytes inside vessel lumen (*), melano-macrophage (dotted arrow). (1000X). **B:** head kidney from a fish exposed to Cd showing an evident thickening of the vein wall (VW), the recruitment of basophils near blood vessels (black arrows) and the loss of the normal cytoarchitecture of the cellular parenchyma (1000X).

Control head kidneys showed a compact homogeneous parenchyma. Large lymphocytes, little lymphocytes, plasma cells, monocytes, melano-macrophages and red cells were observed. Granulocytes, lymphocytes and erythrocytes are 70±17 % of immature cells. The basal endothelial membrane was 1.87±0.32 µm wide and its integrity was maintained (Fig. 1A).

The most prominent features of the damage to the head kidneys of *C. macropomum* exposed to chronic levels of Cd were:

1. Depletion of the hematopoietic precursors (Table 1); 2. Thickening of the basal endothelial membrane, 35% thicker than that of the controls (Table 1); 3. The recruitment of basophils near blood vessels; 4. A disruption of tissue integrity: debris could be observed within cells and there were empty spaces between cells (Fig. 1B).

**Table 1:** Tickness of basal membrane of vessel and cell count in head kidney of *C. macropomum* Cd-exposed.

Parameters	Control	Cd exposed
Tickness of basal membrane (µm)	1.87±0.32	5.35±0.61***
Precursor cells (%)	70±17.04	29±4.36*

*** (*P*<0.001);

* (*P*<0.05).

**Fig. 2:**
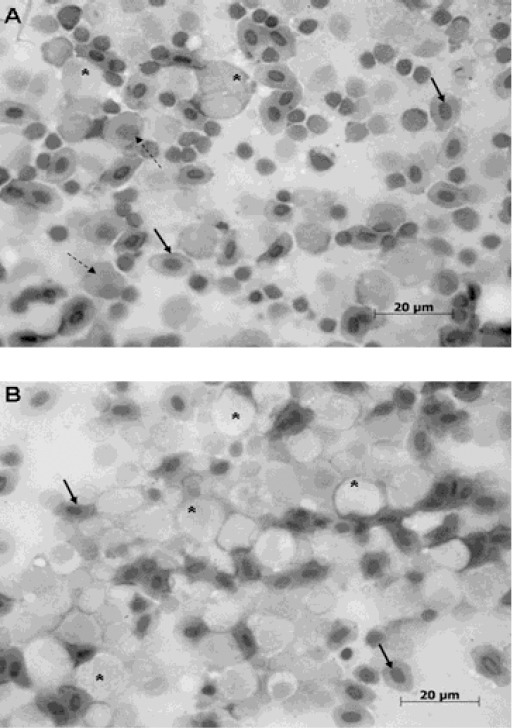
Head kidney imprints of *C. macropomum*. **A:** control imprint with immature granulocytes (*), immature erythrocytes (dotted arrows) and mature erythrocytes (continuous arrows). **B:** imprint of cadmium-exposed kidney showing precursor cells with evident swelling and eccentric nuclei (1000X).

Control head kidney imprints showed both granulocyte-aggregates and erythrocyte-aggregates. Immature granulocytes were large and contained eccentric nuclei with relaxed chromatin. Eosinophilic hyaline granules could be observed inside the cytoplasm, and immature erythrocytes showed a cytoplasm without granules or central nuclei (Fig. 2A).

Cd-exposed fish imprints showed hematopoietic precursors with different degrees of hypertrophy and vacuolization.

Granulocytes were swollen and vacuolization and pinocytosis could be observed; many erythrocyte precursors had eccentric nuclei indicating an aging cell (Fig. 2B).

Cd accumulation in *C. macropomum* kidneys was lowest in the head kidney, where hematopoietic tissue dominates, and greatest in the posterior tail sections, where filtration tissues dominate (Table 2).

**Table 2:** Cadmium concentrations in the head kidney and trunk kidney of *C. macropomum*.

Tissues	Cd concentration (μg.g -1)	
	Control	Cd-exposed
Trunk kidney	0.027±0.0274	5.39±1.8755***
Head kidney	0.004±0.0015	0,018±0,0004***

*** (*P*<0.001).

As a consequence of the decrease in the number of precursor cells in the head kidney, the total blood leukocyte count and differential blood leukocyte count decreased. In contrast, the normal erythrocyte count found in the blood of Cd-exposed fish may be explained by the fact that erythrocytes could be produced by other organs such as the trunk kidney and liver (Table 3).

**Table 3:** Hematological parameters of Cd-exposed fish *C. macropomum*

Parameters	Control	Cd-exposed
Hb (g/dl)	7.31±1.79	5.40±1.32*
Hto (%)	21.03±3.17	18.86±6.30 ns
Erythrocytes (x10^6^mm^3^)	3.54±1.11	3.23±3.95 ns
MCV (fl)	531±150	370±125ns
CHCM (%)	33.86±2	28,08±1,9ns
Leukocytes (x10^4^mm^3^)	3.36±1.81	1.54±0.60**
Granulocytes (Cel/mm^3^)	992,00±63,68	465,00±24,40***
Lymphocytes (Cel/mm^3^)	2353,00±48,70	1065,00±21,62***

ns= no significant

** (P<0.01);

*** (P<0.001)

## Discussion

The median lethal concentration (LC_50_ 96 hours) can be considered very high if we take into account that this metal has been reported as highly toxic and non-bio essential (Sadiq, 1992).

Unlike here, reports in other species of freshwater fish, such as Oreochromis mossambicus, has determined that the Cd 96-h LC_50_ was 11.99 ppm (James, 2000) and Cd CL_50_-96h to *Cichlasoma dimerus* was 44 mg/L (Hirt and Domitrovic, 2002).

The results regarding 96 hour LC_50_ in cachama suggest that these fish are able to tolerate high concentrations of Cd. In natural environments, cachamas from apparently uncontaminated, moderate concentrations of Cd are reported (Salazar-Lugo, 2009).

We did not observe a nephron in the parenchyma, although lymphocyte-aggregate and granulocyte-aggregate regions were found, suggesting that the head kidney in *C*. *macropomum* is an important organ for hematopoiesis.

The observed features of the damage to the head kidneys suggest the involvement of the organ in an inflammatory process. Studies have shown that Cd, at relatively low sub lethal concentrations, can target vascular endothelial cells at a variety of molecular levels, including cell adhesion molecules, metal ion transporters and protein kinase signaling (Woods *et al.*, 2008).

Cell loss has been reported as a typical characteristic of the chronic effects of Cd on the kidneys in animals (Wang *et al.*, 2009; Sinha *et al.*, 2009). On the other hand, the increase in the number of mast cells in various tissues and organs of teleosts seems to be linked to a wide range of stressful conditions, such as exposure to heavy metals (Cd, copper, lead and mercury) and exposure to herbicides and parasitic infections (Manera *et al.*, 2011). In *Sparus aureata*, prolonged exposure to Polychlorinated Biphenyls (PCBs) caused a significant increase in the number of mast cells in connective tissue close to blood vessels of the muscularis layers of the intestine and in regions of active inflammatory responses (Lauriano *et al.*, 2012).

The pattern of Cd accumulation in *C. macropomum* kidneys is similar to that reported by Woodling *et al*. (2001) in wild brown trout *Salmo trutta* and by Kondera *et al*. (2013) in *Cyprinus carpio*. Cd accumulation in Trunk kidney is well-known in fish (Singh *et al.*, 2008, Rauf *et al.*, 2009, Dugmonits *et al.*, 2013). Cd is delivered to kidney by Cd-Metallothionein (Cd-Mt) complexes to eliminate in the urine, however, these complexes Cd-Mt can be retained in this organ for a long period of time (Tang *et al.*, 1998).

A decrease in the number of precursor cells in the head kidney suggests that Cd affects hematopoiesis, especially leucopoiesis, during the early stages of cellular differentiation. Exposure to Cd also reduced the hematopoietic potential of the common carp *Cyprinus carpio*, measured as the ratio of proliferating to apoptotic precursor cell frequency (Kondera and Witeska, 2013).

To summarize, chronic Cd exposure in the freshwater fish *C. macropomum* produced an anomalous head kidney structure, and induced an inflammatory process in this organ, affecting hematopoietic cell differentiation, especially with regard to granulocytes and perhaps affecting its function.
